# Features of the Antitumor Effect of Vaccinia Virus Lister Strain

**DOI:** 10.3390/v8010020

**Published:** 2016-01-12

**Authors:** Evgeniy Zonov, Galina Kochneva, Anastasiya Yunusova, Antonina Grazhdantseva, Vladimir Richter, Elena Ryabchikova

**Affiliations:** 1 Institute of Chemical Biology and Fundamental Medicine, Siberian Branch of the Russian Academy of Sciences (ICBFM SB RAS), 8 Lavrentiev Avenue, Novosibirsk 630090, Russia; zoman89@gmail.com (E.Z.); pretty_lier@mail.ru (A.Y.); vrichter@ngs.ru (V.R.); 2 State Research Center of Virology and Biotechnology “Vector”, Koltsovo 630559, Russia; g.v.kochneva@yandex.ru (G.K.); gaa@vector.nsc.ru (A.G.)

**Keywords:** vaccinia virus, genetically unmodified L-IVP strain, human A-431 carcinoma xenografts, murine Ehrlich carcinoma, VACV-induced arrest of mitoses

## Abstract

Oncolytic abilities of vaccinia virus (VACV) served as a basis for the development of various recombinants for treating cancer; however, “natural” oncolytic properties of the virus are not examined in detail. Our study was conducted to know how the genetically unmodified L-IVP strain of VACV produces its antitumor effect. Human A431 carcinoma xenografts in *nude* mice and murine Ehrlich carcinoma in C57Bl mice were used as targets for VACV, which was injected intratumorally. A set of virological methods, immunohistochemistry, light and electron microscopy was used in the study. We found that in mice bearing A431 carcinoma, the L-IVP strain was observed in visceral organs within two weeks, but rapidly disappeared from the blood. The L-IVP strain caused decrease of sizes in both tumors, however, in different ways. Direct cell destruction by replicating virus plays a main role in regression of A431 carcinoma xenografts, while in Ehrlich carcinoma, which poorly supported VACV replication, the virus induced decrease of mitoses by pushing tumor cells into S-phase of cell cycle. Our study showed that genetically unmodified VACV possesses at least two mechanisms of antitumor effect: direct destruction of tumor cells and suppression of mitoses in tumor cells.

## 1. Introduction

The vaccinia virus (Poxviridae family) is among the most “famous” viruses; it is primarily known for its successful use in vaccination and its role in smallpox eradication [1]. Vaccinia virus (VACV) possesses many unique properties that place this virus at a leading position in molecular biology and genetic engineering. Its ability to kill cancer cells is one of the fundamental biological properties of VACV, and was first reported by Levaditi C. and Nicolau S. in 1923 in the Annals of the Pasteur Institute [2]. Subsequent studies confirmed the oncolytic activity of VACV [3]; however, over the next few decades researchers failed to achieve stable results in treatment of cancer patients using VACV, and most importantly, to avoid complications caused by introducing the infectious virus into human organism. The interest to studies of VACV application in oncology seemed to wane. However, progress in genetic engineering now enables researchers to change the biological properties of VACV over a wide range, and this has led to a surge of interest to oncolytic abilities of genetically modified VACV. Modern studies exploit many properties of VACV in order to reach its full oncolytic potential. These properties include the VACV’s ability to infect a wide range of eukaryotic cells [4], and to actively replicate in the cytoplasm and thus spread among neighboring cells to infect and kill them. Most importantly for genetic engineering are the absence of VACV integration with the host genome and the possibility to insert up to 25,000 base pairs into the viral genome without loss of infectivity [5,6,7]. To enhance VACV oncolytic properties, various genes are inserted: cytokines [8,9,10,11], inhibitors of angiogenesis [12,13], enzymes that convert non-toxic precursors within tumors in their cytotoxic derivatives [14,15], and genes of apoptosis-inducing proteins [16,17]. Detailed reviews and analysis of studies with genetically modified oncolytic VACV, including some which are in clinical trials, recently were published in several comprehensive reviews [7,18,19].

Advances in development of genetically modified VACV variants overshadowed the question: how original parental VACV implements oncolytic (antitumor) effect? We think that it is necessary to know “native” antitumor properties of the parental VACV in order to understand the mechanisms of recombinant viruses’ oncolytic activities and to evaluate the real effects of the genetic modifications. The aim of this work was to study antitumor properties of the VACV strain L-IVP (Lister-Institute of Vaccine Preparations, Moscow, Russia, GenBank accession number: Bank It1780508 LIVP KP233807). It is believed that Lister strain of VACV was isolated in Cologne (Germany) at the Institute of Vaccines in 1870 from a soldier suffering from smallpox, and most probably the strain appeared after the additional infection of a soldier with the laboratory vaccine strain [1].

The Lister strain was used in the program to fight smallpox in the United Kingdom since 1892 and in the Lister Institute (France) since 1916. After transfer to other laboratories, this strain was also known as Liverpool, Merieux 37 and Nigeria. The Lister strain also was transferred to the Institute for Viral Preparations (Moscow, USSR) and used under the acronym L-IVP for vaccination against smallpox and for scientific research [17,20,21]. Many studies have shown the advantages of genetically modified Lister strain as an oncolytic agent in comparison with other VACV strains and other viruses [22].

Our study of apoptin-producing recombinant obtained using L-IVP strain in comparison with the parental strain revealed rapid destruction of human carcinoma A431 xenografts in *nude* mice after intratumoral injection of both viruses [20]. The L-IVP strain clearly demonstrated oncolytic effects via direct destruction of tumor cells (signs of inflammatory reactions and leukocyte accumulation in tumor tissue, and viral destruction of blood vessels were not observed). The question arose: what are other mechanisms may contribute to the antitumor effects of the VACV?

In this study, we examined antitumor effect of the L-IVP strain using murine Ehrlich carcinoma in C57Bl mice and compared that with oncolytic effect of this virus in human A431 carcinoma xenografts in *nude* mice. In contrast with human cells, murine cells are not naturally susceptible to VACV, so it was interesting to compare viral antitumor effects in these two models. Our study showed that the L-IVP strain of VACV possesses antitumor activity towards murine tumor, which is mainly related with mitotic arrest in murine tumor cells.

## 2. Materials and Methods

### 2.1. Virus and Cells

The L-IVP strain of VACV was obtained from the State Collection of Viral and Rickettsial Disease Agents of the State Research Center of Virology and Biotechnology “Vector” (SRC VB “Vector”, Koltsovo, Russia). The strain was cloned and has been passed 6 times in CV-1 cells and purified by centrifugation in sucrose density gradient (25%–45%). The viral preparation was sonicated and titrated using the plaque formation assay in CV-1 cell monolayers. Virus titers were expressed as plaque forming units (PFU) per mL. The viral stock represented 10^9^ PFU/mL in sterile saline and aliquots were stored at −80 °C. Human cancer cell lines (A549, A431, C33A, U87MG, RD, DU145, MCF7, Mel8, SW480, HeLa) of different origin were grown in DMEM (Invitrogen, Waltham, MA, USA) supplemented with 10% fetal calf serum (FCS, HyClone, Logan, UT, USA). Diploid human embryonic LECH-240 cells were grown in F-12 medium (Invitrogen) supplemented with 10% fetal calf serum (FCS, HyClone). MCF10A cells were grown in a specialized culture medium for mammary epithelial cells MEGM Bullet Kit (Lonza, Allendale, NJ, USA).

### 2.2. Cytotoxic Activity of VACV Strain L-IVP toward Human Tumor Cell Lines

Cytotoxic activity of VACV strain L-IVP toward human tumor cell lines was evaluated by XTT microassay (using 2,3_bis_(2-methoxy-4_nitro-5-sulfophenyl)-2H-tetrazolium-5-carboxanilide, Sigma-Aldrich, St. Louis, MO, USA) in 96-well plates (Greiner, Pleidelsheim, Germany) [8]. This method employs the fact that mitochondrial dehydrogenases can convert soluble XTT into formazan, which crystallizes within the cell. Formazan can be solubilized by phenazine methosulfate (PMS) treatment, and the optical density of the solution determined by spectrophotometry accurately reflects the changes of formazan quantities in viable cells. The specific rate of cell death in infected cultures was assessed in relation to uninfected control cells (100% viabilityπ). Cytolytic activity was evaluated as the 50% cytotoxic dose (CD_50_), that is, the virus concentration causing death of 50% of cells. To determine CD_50_, cells growing in a 50% monolayer were infected with sequential tenfold dilutions of viral suspension in 100 μL of 199 medium supplemented with 2% FCS (0.001 to 10 PFU/cell). Following 72 h incubation at 37 °C;, in an atmosphere of 5% CO_2_ and 85% humidity, 50 μL of XTT/PMS mixture were added to each well (the mixture was prepared with 20 μL of 1.25 mM PMS (Fluka, St. Louis, MO, USA) per 1 mL of 1 mg/mL XTT working solution). Plates were incubated for additional 4 h, and optical densities (*ОD*490/620) were determined using the SpectraCount Plate Reader photometer (Packard, Missouri, TX, USA). To obtain a curve that represents the dependence between *OD* and multiplicity of infection, data obtained from five replicates were used to calculate the mean and SD values for each point corresponding to different virus concentrations. This curve was used to determine CD_50_ as the virus concentration at which the *ОD*490/620 value of infected wells was 50% of *ОD*490/620 measured in wells with uninfected culture. CD_50_ values were compared across cell cultures; higher CD_50_ indicated the lower oncolytic activity of the given virus strain in a particular cell culture. All computations were performed using LabView software.

### 2.3. Animal Studies

Studies with mice were performed under protocols approved by the SRC VB VECTOR Institutional Animal Care and Use Committee (NIH Office 85 of Laboratory Animal Welfare, Number A5505-02).

**A431 human carcinoma model.** Female *Nu/Nu* mice (Nursery for Laboratory Animals, Institute of Bioorganic Chemistry, Moscow, Russia) were used for the xenograft model of human A431 carcinoma. Tumors were generated in 8–10 week old *Nu/Nu* mice (20–26 g) by subcutaneous injections of 5 × 10^6^ A431 cells (in 100 mL PBS) in the left hind region. Tumors were measured in two dimensions using a digital caliper, and their volume was calculated by the following formula: [length × width^2^ × 0.5] [20]. When tumors reached 200–250 mm^3^ in volume (10 days after tumor cells injection), the mice received a single injection of the LIVP strain (1 × 10^7^ PFU/mouse in 100 mL of saline) into tumor. The control group of mice received 100 mL of saline.

To assess the dynamics of virus accumulation in tissues and organs, mice were sacrificed every 48 h in the period from 2 to 20 days after injection; and every 96 h in the period from 20th to 55th days (two mice at each time-point from each experimental group). Specimens of xenografts, spleen, liver, lungs, kidneys and blood were collected from two mice from experimental and control groups at each time point. To evaluate viral content, homogenates of organs and tissues (10%, *v*/*v*) were prepared in saline, sonicated, centrifuged and then titrated by plaque formation assay on a monolayer of CV-1 cells. For microscopic studies xenografts were collected from mice of each control and infected groups at 2, 4 and 8 days after VACV injection. Xenografts of infected mice also were sampled on 36th and 55th days after virus injection. The tumors and adjacent tissues were dissected and fixed in 4% paraformaldehyde solution.

**Murine Ehrlich carcinoma model.** Male mice C57Bl were inoculated subcutaneously with murine Ehrlich ascites carcinoma cells (13 × 10^6^ in 0.4 mL of sterile saline) in the thigh right hind paw. After 7 days, when tumors reached 200–220 mm^3^ in volume, mice received a single injection of the L-IVP stain (1 × 10^7^ PFU/mouse in 100 mL of sterile saline) into tumor. Mice of the control group received 100 mL of saline. Mice were sacrificed on days 2, 4, 6, 8, 12 and 14 after virus injection (three mice at each time-point from each group); volume of their tumors was measured as described above. Tumors were dissected and fixed in 4% paraformaldehyde solution.

Female C57/B1 mice were injected intraperitoneally with 3–3.5 × 10^6^ cells of Ehrlich carcinoma cells in 0.2 mL of saline. Four days after cell injection 15 mice received intraperitoneally 1.3 × 10^7^ PFU of L-IVP strain in 100 mL of saline, and 10 mice were injected with 100 mL of saline (control). Mice were sacrificed on days 3, 6, 9 and 10 after VACV injection (three mice from at each time-point from each group), abdominal cavity was opened and ascitic fluid was collected. Some fluid (0.3–0.4 mL) was used for VACV titration by plaque formation assay on a monolayer of CV-1 cells. The remaining ascitic fluid was cleaned by three repeats of dilution with saline and centrifugation at 2000 RPM for 5 min. Final pellets were fixed in 4% paraformaldehyde solution.

We attempted to propagate cells of Ehrlich carcinoma *in vitro* without success; in agreement with published studies, the tumor was only able to be grown *in vivo*.

### 2.4. Microscopic Studies

For histologic studies, specimens of tumors and ascitic cells were routinely processed using Sakura Tissue-TEK II device (Sakura Finetek, Tokyo, Japan) and embedded in Histomix. Paraffin sections were stained with hematoxylin and eosin. The sections for immunohistochemistry were mounted on polylysine-coated slides (Thermo Scientific, Waltham, MA, USA). Rabbit polyclonal antibodies (Abcam, Cambridge, UK) in concentrations 2.5 and 5 mg/mL were applied to detect proteins Apaf-1 (a marker of apoptosis) and Ki-67 (proliferation marker). The cell cycle S-phase marker, PCNA, was detected using corresponding mouse antibodies in concentration 5 mg/mL (BioLegend, San Diego, CA, USA). Rabbit polyclonal antibodies against CD3 and CD11b (Abcam) were used to detect T-lymphocytes or monocytes and granulocytes on paraffin sections of solid Ehrlich carcinoma correspondingly. The antibodies were diluted in accordance with manufacturer’s recommendations. The antigens were visualized using AEC Single Solution detection kit (Abcam) in accordance with the manufacturer's recommendations. Paraffin sections were examined using Leica DM 2500 (Leica, Wetzlar, Germany) microscope supplied with digital camera Leica DFC420 C (Leica).

For electron microscopy, fixed in 4% paraform samples of xenografts and ascitic cells were postfixed in 1% osmium tetroxide solution, then routinely processed and embedded into a mixture of epon-araldite (EMS, Hatfield, PA, USA). Semithin sections were prepared from hard blocks, stained with Azur II and examined in light microscope for selection of the areas for ultrathin sectioning and counting mitotic cells. Ultrathin sections were cut using Leica EM UC7 ultramicrotome (Leica), routinely contrasted by uranylacetate and lead citrate (SPI, West Chester, PA, USA) and examined in transmission electron microscope JEM 1400 (Jeol, Tokyo, Japan). Images were collected using the side-mounted digital camera Veleta (Olympus Corporation, Tokyo, Japan).

Tumor structures on paraffin sections were measured using Axio Vision SE64 Rel. 4.9.1 software. The number of mitotic cells was calculated at ×400 magnification in 10 randomly selected fields in peripheral zone of A431 carcinoma. In paraffin sections of Ehrlich ascitic carcinoma the amount of mitotic and immunohistochemically positive cells were counted in randomly selected fields, not less than 2000 cells were used for each calculation.

Statistical analysis was performed using STATISTICA 8.0.360.0 [23]. The significance of differences was assessed by Mann-Whitney-Wilcoxon method (U-Mann-Whitney test).

## 3. Results

### 3.1. Cytolytic Activity of VACV L-IVP Strain for Human Tumor Cell Lines in Vitro

**Figure 1:**
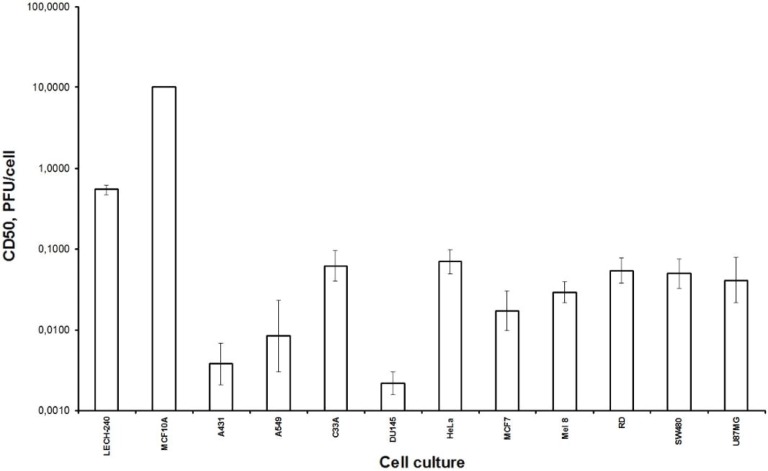
Cytotoxic activity of vaccinia virus (VACV) strain L-IVP in human cells. Ten tumor cell lines were used: A549, A431, C33A, U87MG, RD, DU145, MCF7, Mel8, SW480 and HeLa. Diploid human embryonic LECH-240 and normal breast epithelium MCF10A cells were tested to make a comparison. Cells were grown in 96-well plates, infected with virus doses ranging from 0.001 to 10.0 PFU/cell (sequential tenfold dilutions); 50% cytotoxic dose (CD_50_) was calculated using the XTT test (see Materials and methods). Statistical analysis included results of three independent experiments. Data are presented as the mean ± SD.

For better understanding of the L-IVP strain’s oncolytic abilities, we examined the selectivity of viral lytic effect in normal, diploid and cancer cell lines (Figure 1). We used normal breast epithelium cells MCF10A, diploid human embryonic lung cells LECH-240, and ten cell lines that represent human tumors of different origin: epidermoid carcinoma A431, lung carcinoma A549, cervical cancer cells C33A and HeLa, breast adenocarcinoma MCF7, melanoma Mel 8, rhabdomyosarcoma RD, prostate cancer DU145, epithelial glioblastoma/astrocytoma U87MG and colon adenocarcinoma SW480. As is evident from Figure 1, the L-IVP strain exhibited significantly higher lytic effect on tumor cell lines than on normal or diploid human cells (*p* < 0.05). The data confirm a natural VACV tropism for tumor cells that was noticed earlier [9]. Human cancer cells A431, A549 and DU145 were highly sensitive to the L-IVP strain and demonstrated CD_50_ values 0.004, 0.008 and 0.002 PFU/cell, correspondingly (Figure 1). The selectivity index calculated as the ratio of CD_50_ value for normal MCF10A (>10 PFU/cell) and tumor cells (0.002–0.07 PFU/cell), was more than 100 times higher for all types of cancer cells independently from their origin.

### 3.2. Virus Dissemination in Tissues and Organs of Mice Bearing Carcinoma A431

**Figure 2:**
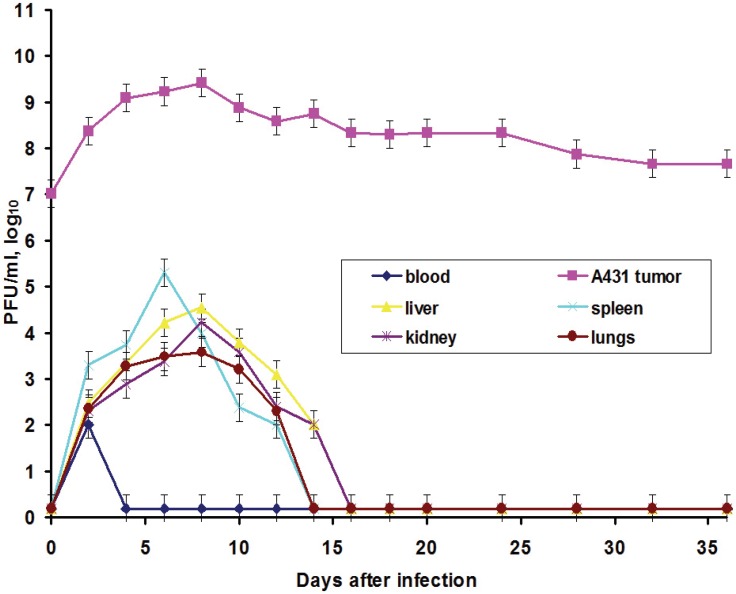
Kinetics of VACV strain L-IVP accumulation in human A431 carcinoma xenografts, blood and visceral organs of *nude* mice after intratumoral virus injection. The bottom line indicates detection limit of the method (about 10 PFU/mL). Data are presented as mean ± SD.

Our previous study [20] revealed high titers of the L-IVP strain in carcinoma A431 xenografts on days 2 and 4 after virus injection: 2.3 × 10^8^ PFU/mL and 1.2 × 10^9^ PFU/mL. The maximum viral concentration in the tumor was reached on day 8 post injection (2.6 × 10^9^ PFU/mL). In order to examine the possibility of VACV’s spread throughout the organism, and thereby to infect cells distant from the primary tumor or metastases, we determined the presence of the L-IVP strain in blood and visceral organs. This ability of VACV is noted in few publications [7], but was not examined directly. We found the virus in small concentration (10^2^ PFU/mL) in blood 2 days after injection and noted the accumulation of the virus in visceral organs of mice within 14 days after intratumoral injection (Figure 2). We suppose that the virus enters the blood in the moment of injection and spreads inside the organism. However, secondary viremia was not registered within 55 days of observation obviously due to the low level of VAVC replication in non-tumor cells and rapid clearance of the virus from mouse organs. Comparison of virus titers in xenografts and visceral organs clearly shows high selectivity of VACV toward tumor cells *in vivo*. The presence of the virus in blood and visceral organs indicates that it is possible for the virus to infect tumor cells from a location somewhere outside of the primary tumor.

### 3.3. Ultrastructural Characteristics of the L-IVP Strain Replication in Cells of A431 and Ehrlich Carcinomas

**Figure 3:**
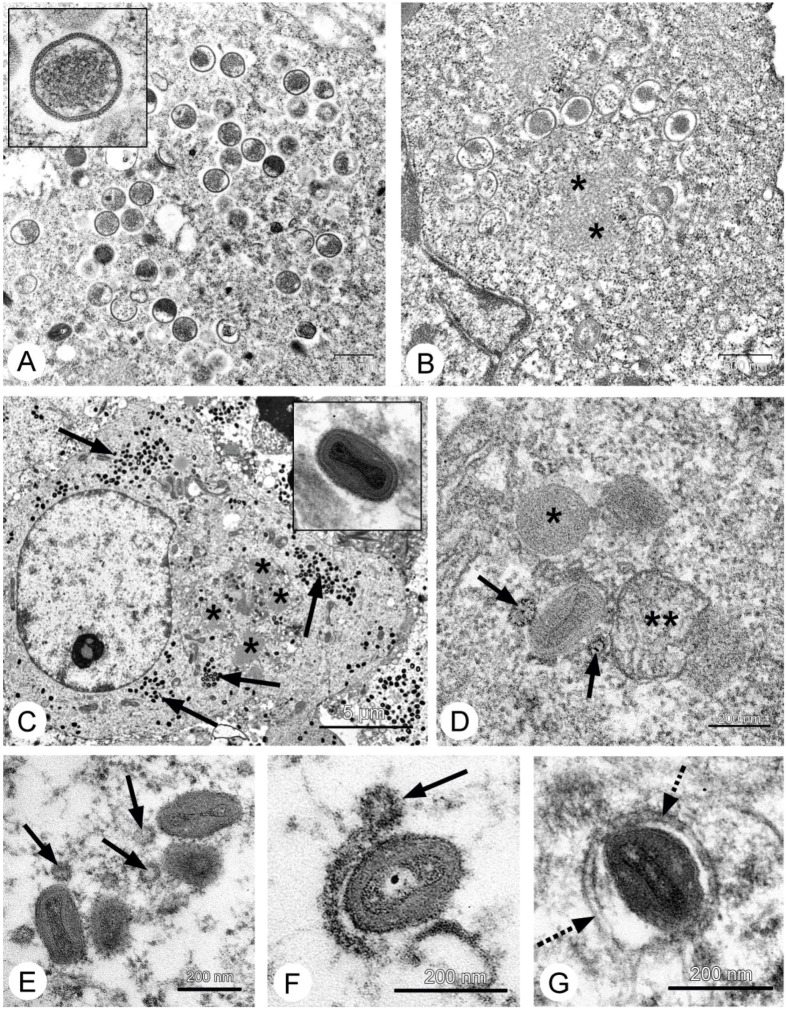
Replication of the L-IVP strain of VACV in cells of human A431 carcinoma (**A**,**C**,**E**–**G**) and murine Ehrlich ascitic carcinoma (**B**,**D**) cells. Photos **A**,**B** show the formation of immature virus particles, the insert in A shows a virion at high magnification, the asterisks show a viroplasm. Photo **C** shows infected cell containing viroplasm (*) and accumulations of mature virions (shown by arrows), the insert for **C** shows a mature virion at large magnification. Photos **D**–**G** show sequential steps of formation of “enveloped” virions. The arrows show Golgi-derived vesicles, which fuse and thereby form double-membrane envelope (shown by dotted arrow). The asterisk on photo **D** shows immature virion, the double asterisks show mitochondria.

Electron microscopy easily found VACV replication in cells of carcinoma A431 xenografts after 2 days post virus injection. Infected cells contained granular viroplasm, immature and mature viral particles (Figure 3). Roundish immature virions (250–300 nm) accumulated in the cytoplasm as loose aggregates about 3 μm in size; 10%–15% of them showed signs of alteration (Figure 3A). Mature oval- or brick-shaped viral particles (250–300 nm) (Figure 3C) formed 2–3 aggregations per section of infected cell. Many “enveloped” virions were observed in cells and extracellular spaces of carcinoma A431 two days after virus injection. The envelope of VACV particles was formed by fused vesicles, which derived from Golgi trans-region (Figure 3E–G). Thus, the viruses of the L-IVP strain actively replicates cells in human carcinoma A431and large number of mature virions accumulate inside them.

In contrast, cells of murine Ehrlich carcinoma are unable to support active replication of the VACV L-IVP strain. Using electron microscopy, we failed to find signs of virus replication in any of the samples of solid Ehrlich carcinoma, including those collected after 9 days post virus injection. Presumably, this reflects a specificity of virus-cell interaction: the mouse is not a natural host for VACV, so murine cells cannot efficiently support the reproduction of this virus.

However, there was distinctive evidence of an antitumor effect (see below) indicating that virus replication could occur in a small number of cells, below the sensitivity of electron microscopy. Electron microscopic examination of Ehrlich carcinoma’s ascitic form revealed small numbers of infected cells (about 1 cell in 10 thousands), corresponding to low titers of the L-IVP strain in ascitic fluid, which varied from 10^2^ to 10^5^ PFU/mL in CV-1 cells. Relatively active virus replication was observed on 9th day after virus injection. Morphological characteristics of the L-IVP strain replication in ascitic Ehrlich carcinoma cells were identical to those in carcinoma A-431 cells, but much fewer viral factories were noted. Thus, viral inclusions were small (1–1.5 mm) and contained few immature viral particles (Figure 3B). The total quantities of viral progeny were incomparably less than in cells of carcinoma A431; few mature virions were scattered in cytoplasm. Although there was a low level of replication, the L-IVP strain has produced “enveloped” virus particles (Figure 3D), which comprised about 30% of total mature virions. About a half of the mature virus particles showed altered morphology: nucleoid and lateral bodies were not seen on ultrathin sections. Thus, the VACV L-IVP strain replicated poorly in cells of murine Ehrlich carcinoma, in contrast to human cells of carcinoma A431.

### 3.4. Antitumor Effect of the L-IVP Strain on Two Carcinomas

**Figure 4:**
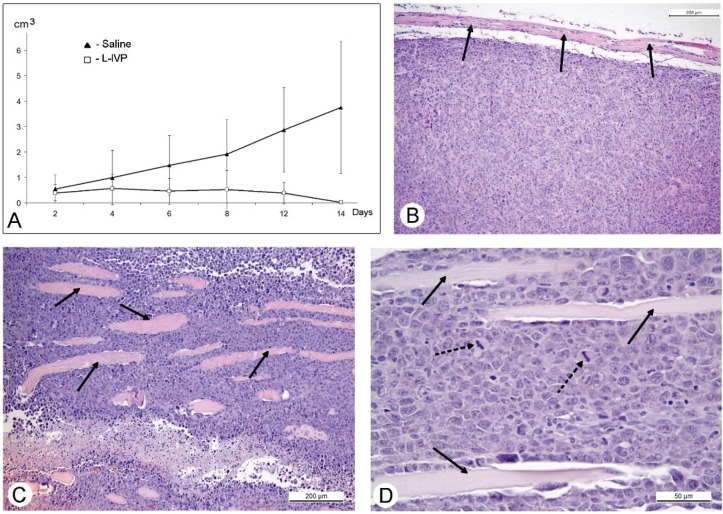
Changes of murine Ehrlich solid carcinoma volume after injection of the L-IVP strain and saline (**A**); period after virus injection is shown on X-axis, tumor volume—on Y-axis. Paraffin section of A431 carcinoma showing capsule (shown by arrows) and smooth outline (**B**); and Ehrlich solid carcinoma (**C**,**D**). Arrows show fragments of muscle tissue, asterisks show necrotic zone, dotted arrows show mitoses. Hematoxyline and eosin staining.

Our previous study showed that injection of the L-IVP strain into human carcinoma A431 xenografts led to the delay and subsequent stopping of their growth, while xenografts injected with saline continued to grow in size. Indeed, on day 55 the tumor volume in virus and saline injected mice differed by more than 6 times. These results led to the conclusion that active replication of the virus provides rapid destruction of the A431 carcinoma [20]. The Ehrlich solid carcinoma tumors injected with the L-IVP strain also decreased in size in comparison with saline-injected control mice (Figure 4). However, when we examined sections of solid Ehrlich carcinoma in the electron microscope, we could not find signs of virus replication. Ehrlich solid carcinoma tumor had distinct outlines and had no capsule, and aggressively invaded into muscles. In contrast, the carcinoma A431 was compact and surrounded by distinct capsule (Figure 4). The structure of Ehrlich solid carcinoma greatly complicated the study and made accurate measurements and calculations of tumor parameters impossible. Nevertheless, comparison of histological sections of virus-injected and saline-injected tumors found no differences in areas of necrotic zones and no signs of tumor destruction caused by VACV replication. We also did not find signs of increased apoptosis of tumor cells.

Obviously, the immune system could contribute to the delay of tumor growth, however, in both carcinomas, there were no accumulations of leukocytes noted in routinely stained paraffin sections and in ultrathin sections. We applied immunohistochemistry to recognize T-lymphocytes (CD3 marker), monocytes and granulocytes (CD11b marker) in carcinoma sections. Examination of carcinoma A431 injected with VACV or saline revealed some CD11b-positive cells in the capsule surrounding the tumor, and single CD11b-positive cells scattered in tumor tissue. The amounts and distribution of monocytes and granulocytes in A431 carcinoma did not differ in saline- and VACV-injected tumors. Cells, positive for CD3 antigen were absent at all in sections of A431 carcinoma, because *nude* mice are deficient in T-lymphocytes.

**Figure 5:**
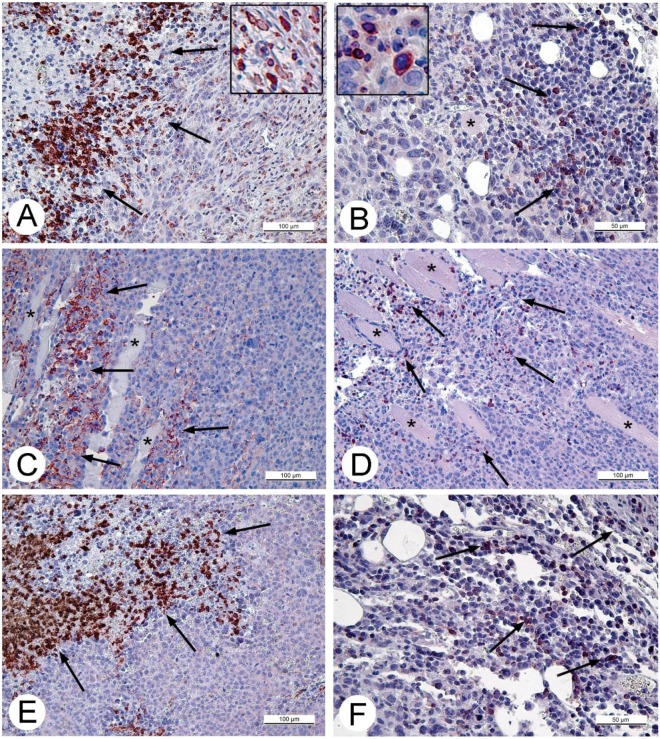
Immunohistochemical detection of monocytes and granulocytes (CD11b antibodies) and T-lymphocytes (CD3 antibodies) in paraffin sections of Ehrlich carcinoma. Upper arrow shows reaction in sections of saline-treated tumors (**A**) CD11b-positive cells; and (**B**) CD3-positive cells, both 14 days after injection. Inserts show positively stained cells at high magnification. CD11b-positive cells in the tumors injected with VACV; (**C**) 6 days and (**E**) 14 days after the injection. Rare T-lymphocytes are seen in in Ehrlich carcinoma (**D**) 6 days and (**F**) 14 days after VACV injection. Positively stained cells are shown by arrows. Asterisks show muscle fibers in Ehrlich carcinoma. Chromogen AEC, counterstained with hematoxyline.

We expected to see evidence of cell immune response in Ehrlich carcinoma because it is murine tumor implanted to immunocompetent C57B1 mice. The same patterns of accumulations of the monocytes and granulocytes at the border of tumor and in necroses (Figure 5C) were found in sections of Ehrlich carcinoma injected with saline (Figure 5A), and in sections of carcinoma, injected with VACV (Figure 5C,E). The T-cells were scarce and mostly were located in connective tissue at the border of the tumor; very few single T-cells were observed in tissue of saline-injected tumors (Figure 5B) and in the VACV-injected tumors (Figure 5D,F). It should be noted, that signs of tumor tissue damage were not found in vicinity of both CD3- and CD11b-positive cells.

The patterns of immunohistochemical staining of saline- and VACV-injected tumors over the whole period of the experiment (14 days) were indistinguishable, indicating that the decrease in tumor size after virus injection is not related to development of virus-induced cell immune response.

### 3.5. Examination of Cell Cycle in Ehrlich Ascitic and A431 Carcinomas

**Figure 6:**
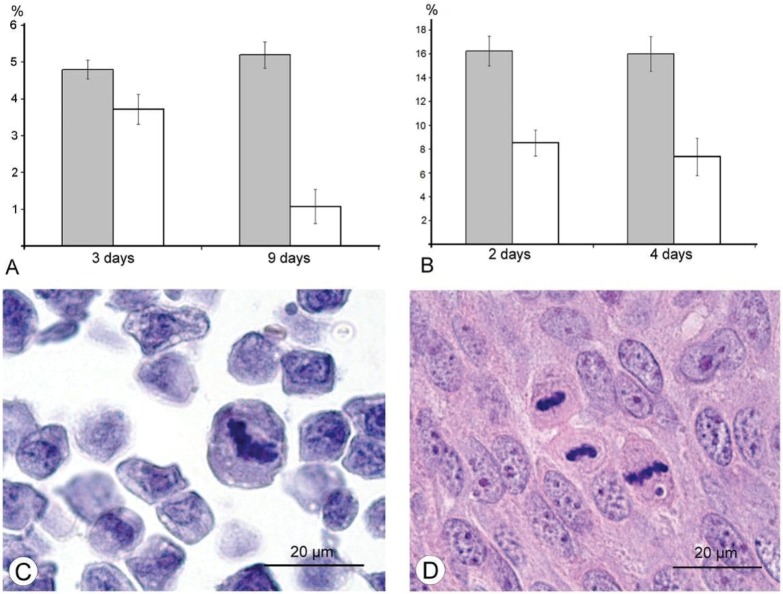
Decrease in number of mitoses in murine Ehrlich ascitic carcinoma (**A**) and human A431 carcinoma xenografts (**B**) after injection of the L-IVP strain. The period after virus injection is shown on the X-axis; the percent of mitotic cells in shown on the Y-axis. The white columns correspond to the L-IVP treated mice, the grey columns correspond to saline treated. Counting was performed on paraffin sections at 400-fold magnification. Photos (**C**) and (**D**) show mitotic cells in paraffin section of Ehrlich ascitic and A431 carcinomas, respectively.

Tumor cells divide extensively, and we examined the possible alterations in this process caused by VACV. A decrease in the amount of mitoses was noted in paraffin and semi-thin sections of Ehrlich solid carcinoma, but the carcinoma’s irregular shape and the presence of muscles made accurate counting of mitoses impossible. To resolve this task and evaluate the cell cycle characteristics, we examined paraffin sections of ascitic Ehrlich carcinoma cells and found a decrease in number of mitotic cells after the injection of L-IVP strain compared with saline-injected tumors (Figure 6A). After this, we examined paraffin sections of human carcinoma A431 on days 2 and 4 after injection of the L-IVP strain and found a decrease in the number of mitotic cells in comparison with saline-injected tumors (Figure 6B). The calculation of the number of mitotic cells in A431 carcinoma at later stages after virus injection was impossible, because the replicating virus destroyed all the tumor cells. The results clearly indicated that L-IVP strain inhibits mitotic division in both mouse Ehrlich and human A431 carcinomas. Details of the changes in mitosis were even more evident in Ehrlich carcinoma because the L-IVP strain did not destroy the tumor tissue.

**Figure 7:**
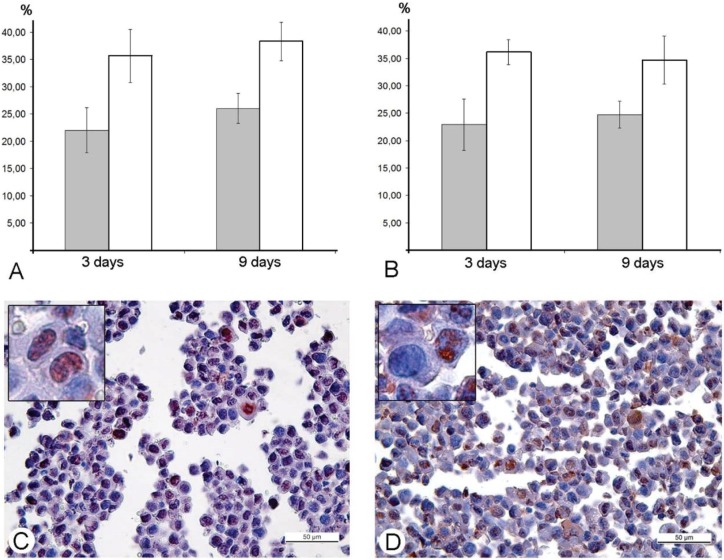
Increase in number of Ki-67 (**A**) and PCNA-positive (**B**) cells in murine Ehrlich ascitic carcinoma in mice treated with the L-IVP strain (white columns) and saline (grey columns). Horizontal line indicates the time after virus injection. Counting was done on paraffin sections, which were stained immunohistochemically at 400-fold magnification. Photos **C** and **D** illustrate immunochistochemical staining for Ki-67 (**C**) and PCNA (**D**) in Ehrlich ascitic carcinoma cells, 3 days after injection of the L-IVP strain and saline, respectively. Chromogen AEC, counterstained with hematoxyline.

For further understanding of how the L-IVP strain affects the cell cycle, we performed an immunohistochemical study of expression of Ki-67 protein, which is widely used in cancer diagnostics as a marker of proliferating cells [24]. We found increase in the quantity of Ki-67-positive cells in sections of Ehrlich ascitic carcinoma (Figure 6A,C), indicating that there is an increase in the amount of proliferating cells. These results led to a proposal that the L-IVP strain causes an imbalance in the cell cycle. Counting of cells positively stained for PCNA (marker of S-phase) [25] revealed an increase in the number of such cells in L-IVP injected Ehrlich ascitic carcinoma (Figure 7B,D). These findings led to conclusion that the L-IVP strain compels the cells to stay in S-phase and thereby reduces number of mitotic cells, which leads to a delay in tumor growth.

There were no changes in number of Ki-67-positive and PCNA-positive cells between saline- and VACV-injected A431 carcinoma xenografts on days 2 and 4 post-injection, obviously, due to dominating role of the virus replication. Thus, the L-IVP strain causes a decrease in the number of mitoses in A431 carcinoma, however, it does not influence number of cells expressing Ki-67 and PCNA proteins.

## 4. Discussion

The antitumor effect of various recombinants of VACV is a subject for hundreds of studies and tens of reviews, but information about antitumor properties of initial, genetically unmodified VACV or “natural” is scarce. We became interested in the features of antitumor effect of genetically unmodified VACV when we studied the antitumor effects of apoptin-expressing recombinant toward A431 human adenocarcinoma xenografts in comparison with parental the L-IVP strain. We found that extensive replication of the L-IVP strain in tumor cells was main factor providing antitumor effect [20]. The present study was conducted to understand better what kind of mechanisms could exploit properties of genetically unmodified VACV in the destruction of the tumors.

Firstly, we examined the lytic effect of the L-IVP strain replication in the cells originating from various types of human tumors in comparison with diploid human cells (Figure 1), and we found high selectivity of the virus strain toward cancer cells *in vitro*. This phenomenon was described earlier [26], and our data fully confirmed this. Localization of the VACV in tumor nodes of experimental animals was shown using GFP-expressing recombinants [13,27,28], and we directly showed selective infection of the tumor cells with the L-IVP strain by electron microscopy: the virus did not replicate in cells of blood vessels and connective tissue [20].

In this work, we also elucidated another important characteristic of the VACV with respect to tumors: the ability of the virus to spread in organism and to be present in visceral organs. Titration of the L-IVP virus using the PFU method revealed the presence of the infectious (living) virus in liver, spleen, lungs and kidneys for two weeks, while the virus rapidly disappeared from blood (Figure 3).

We suppose that virus entered the blood flow in the moment of injection, because the data do not give support or describe the migration of the virus outside the tumor. The titers in the visceral organs increase for some time and their values are higher than maximal value in blood, evidencing for the replication of the L-IVP strain in cells of the examined organs. Release of viral progeny from infected cells and infection of distant cells was considered as advantageous feature for the realization of the VACV oncolytic effect [7,18,29]. Replication of the L-IVP strain in mouse visceral organs illustrates ability of virus to reach distant targets and demonstrates the possibility of the virus to infect tumor cells distant from primary virus injection site, for example in metastases.

The main goal of this work was to study the VACV antitumor effect in mice with non-compromised immune system using a murine tumor, because the human carcinoma A431 xenografts in *nude* mice represents an artificial experimental system. We used murine Ehrlich carcinoma, which can grow in solid and ascitic forms in different lines of mice [30,31]. The protocol of this study was similar to those of A431 carcinoma, and similar to those, we observed a decrease in volume of the solid tumor in comparison to saline-treated control (Figure 4). However, in contrast to A431 carcinoma, we failed to find signs of virus replication in the tumor using electron microscopy, although we examined numerous sections in all virus-injected animals. This could be explained by a small amount of infected cells, which is below the detection capabilities of the method. Examination of paraffin sections of Ehrlich solid carcinoma revealed no signs of selective destruction and apoptosis in VACV-injected tumors compared with saline-injected during the whole period of observations.

Solid tumors, growing in living organism, reach the stage when their supply with oxygen and nutrients does not keep pace with the growth of a tumor, leading to formation of necroses. This process promotes entry of tumor tissue antigens in blood and development of immune response to tumor [32]. Obviously, CD11b- and CD3-positive cells in solid Ehrlich carcinoma found after 9 days post tumor transplantation (2 days after injection of saline), represent development of mouse immune response to the carcinoma. However, the differences in localization and numbers of CD11b- and CD3-positive cells between saline- and VACV-injected mice were not detected within 14 days of the experiment. We suppose that the duration of the experiment (14 days, time of the death of mice in control group) was too short for the development of detectable signs of cell immune response to the VACV in the Ehrlich carcinoma. The immune response to oncolytic viruses is a complicated event, unfolding at the background of tumor evading the immune system using various mechanisms including damage of antigen presentation and induction of immune tolerance [33,34]. In addition, the environment in the tissue of a tumor is immunosuppressive [35]. All these circumstances could contribute to a delay in the development of virus-induced cell immune response in the Ehrlich carcinoma.

Therefore, we could not understand how the L-IVP strain causes the decrease in volume of Ehrlich solid carcinoma. We proposed that the virus replicates in small number of tumor cells and releases some substances altering the tumor growth. To test this, we examined the effect of L-IVP strain on ascitic form of Ehrlich carcinoma, which allows one to obtain homogeneous preparations of tumor cells. As we expected, replication of the L-IVP strain in carcinoma cells was weak (the titers did not exceed 10^5^ PFU/mL), and electron microscopy revealed few infected cells.

The results of our study excluded direct destruction of tumor cells by VACV replication and immune response as possible factors for the decreasing sizes of Ehrlich carcinoma. The ability of the VACV to reduce amount of mitotic cells in a tumor was noted more than 60 years ago [36], and we supposed that this could play a role in decrease of tumor size after injection of VACV in our experiments. Indeed, a comparison of number of mitoses counted in the L-IVP- and saline-treated carcinomas revealed that the decrease occurs after treatment with the virus (Figure 6). The same effect of the L-IVP strain was observed in A431 carcinoma xenografts on days 2 and 4 after the L-IVP strain injection, however their rapid destruction by VACV do not permit to observe this effect longer than 4 days (Figure 6A).

It would have been interesting to follow this antimitotic effect of VACV on the same tumor cells *in vitro*, however, Ehrlich carcinoma is unable grow *in vitro*. The cells of A431 carcinoma are highly sensitive to VACV and are destroyed too quickly to observe this effect. The effect of VACV on mitoses of tumor cells was observed about 50 years ago; authors used different viral strains and cell cultures, and various experimental designs and methods, these showed that antimitotic effect of the virus could present in both tumor and non-tumor cells [37,38,39,40]. Recent studies of VACV influence on cell cycle are devoted to molecular mechanisms of VACV interaction with a cell, and showed that the virus shifts the cell to S-phase and thereby provides more efficient viral replication. This phenomenon was shown to be related with hyperphosphorylation of p53 by viral early B1R kinase, leading to p53 downregulation, which, in turn, could promote synthesis of DNA [40,41].

Na-Kyung Yoo and co-workers, 2008 [42] examined the shifting of VACV infected cells to S-phase using recombinant vTF7-3 VACV and rapidly proliferating human osteosarcoma 143B and HeLa cells. The results showed that VACV possesses a unique cell-cycle control mechanism, involving inactivation of p53 and Rb, which are associated with the RNA polymerase III transcription factor B (TFIIIB) subunits, TBP and Brf1, respectively. All the examined events unfold inside infected cells *in vitro*. Our study was performed in animal models, and immunohistochemical reaction for the PCNA-antigen (marker of S-phase) showed that the amount of the cells in the S-phase is incomparably larger, than the amount of infected cells in Ehrlich ascitic carcinoma. One of the possible explanations of damage of cell cycle is blockage of VACV replication in the early stages, when viral early B1R kinase is expressed, but visual signs of the virus replication are not seen. In such case, we will not see infected cells, and viral yield will be low, just as we observed. It is interesting that the ability of the virus to promote entry of infected cells into S-phase is considered as advantageous mechanism, providing more efficient replication of VACV and many other DNA- and RNA-viruses [43]. Our study showed another facet of this ability—the stopping of mitotic division of tumor cells resulting in delays of tumor growth. Usage of mouse Ehrlich carcinoma in mice demonstrated this effect clearly, but pointed new questions about its mechanisms. One of these questions is what is possible influence of immune system mediators induced by VACV on mitotic division of tumor cells. However, decrease in amount of mitoses was evident from day 2, while specific immune response to a virus develops later.

Our study showed that VACV achieves its antitumor abilities in both A431 and Ehrlich carcinomas; however, this antitumor activity is achieved in different ways, presumably depending on the tumor origination. Humans are a natural host for VACV, and cells of human A431 carcinoma support active replication of the virus, which rapidly destroys tumor cells. In this case, the influence of the VACV infection on cell cycle recedes into the background, and is overwhelmed by the complete destruction of the tumor. A mouse is not naturally susceptible to VACV, so it was difficult to expect high destructive action of the virus on the tumor, and our research showed this. Despite of poor replication, VACV demonstrated an antitumor effect, and our study revealed that this effect is associated with accumulation of tumor cells in S-phase, which reduces number of dividing cells and, correspondingly, delays tumor growth. The susceptibility of the tumor cells to the virus determines predominance of first or second mechanism. The VACV is “a perfect parasite” utilizing all cellular mechanisms for successful survival, and a clear understanding of how the virus can alter undesired cells could be useful for development of effective anticancer therapeutics.

## 5. Conclusions

In this study, we showed that genetically unmodified, “natural” VACV achieves its antitumor effect not only by direct destruction of infected tumor cells, but also by arresting mitotic division of tumor cells in the S-phase. The latter provides an antitumor effect in murine model, which is not naturally susceptible to VACV, and thereby confirms high oncolytic potential of the VACV.
